# Longitudinal Performance of Senescence Accelerated Mouse Prone-Strain 8 (SAMP8) Mice in an Olfactory-Visual Water Maze Challenge

**DOI:** 10.3389/fnbeh.2018.00174

**Published:** 2018-08-28

**Authors:** Virginie Lam, Ryusuke Takechi, Matthew A. Albrecht, Zachary John D'Alonzo, Liam Graneri, Mark J. Hackett, Stephanie Coulson, Nicholas Fimognari, Michael Nesbit, John C. L. Mamo

**Affiliations:** ^1^Curtin Health Innovation Research Institute, Faculty of Health Sciences, Curtin University, Perth, WA, Australia; ^2^School of Public Health, Faculty of Health Sciences, Curtin University, Perth, WA, Australia; ^3^School of Pharmacy and Biomedical Sciences, Faculty of Health Sciences, Curtin University, Perth, WA, Australia; ^4^School of Molecular and Life Sciences, Faculty of Science and Engineering, Curtin University, Perth, WA, Australia

**Keywords:** morris water maze, olfactory sense, cognition, murine model, SAMP8 strain

## Abstract

Morris water maze (MWM) is widely used to assess cognitive deficits in pre-clinical rodent models. Latency time to reach escape platform is frequently reported, but may be confounded by deficits in visual acuity, or differences in locomotor activity. This study compared performance of Senescence Accelerated Mouse Prone-Strain 8 (SAMP8) and control Senescence Accelerated Mouse Resistant-Strain 1 (SAMR1) mice in classical MWM, relative to performance in a newly developed olfactory-visual maze testing protocol. Performance indicated as the escape time to rescue platform for classical MWM testing showed that SAMP8 mice as young as 6 weeks of age did poorly relative to age-matched SAMR1 mice. The olfactory-visual maze challenge described better discriminated SAMP8 vs. SAMR1 mice than classical MWM testing, based on latency time measures. Consideration of the distance traveled rather than latency time in the classical MWM found no treatment effects between SAMP8 and SAMR1 at 40 weeks of age and the olfactory-visual measures of performance confirmed the classical MWM findings. Longitudinal (repeat) assessment of SAMP8 and SAMR1 performance at 6, 20, 30, and 40 weeks of age in the olfactory-visual testing protocol showed no age-associated deficits in SAMP8 mice to the last age end-point indicated. Collectively, the results from this study suggest the olfactory-visual testing protocol may be advantageous compared to classical MWM as it avoids potential confounders of visual impairment in some strains of mice and indeed, may offer insight into cognitive and behavioral deficits that develop with advanced age in the widely used SAMP8 murine model.

## Introduction

The Morris-Water-Maze (MWM) is a widely used visual-spatial learning technique of learning and memory with a strong dependence on hippocampal integrity, synaptic plasticity and NMDA receptor function (Vorhees and Williams, 2006). The MWM is based on the mouse utilizing extra-maze cues (i.e., fixtures, or images positioned exterior to the water-maze tank), to help orientate itself and locate a rescue platform submerged beneath the surface of the water. Prior to performance acquisition measures in MWM, each mouse is repeatedly trained for up-to 10 times over several days to learn to find the rescue platform. During training, the rescue platform remains visible and is positioned slightly above the water within a fixed quadrant of the circular pool. Thereafter, for the acquisition phase trialing, the rescue platform is slightly submerged beneath the water within the same quadrant position as during training. Acquisition performance therefore represents recall-memory of the proximity of where rescue ordinarily occurs. Most studies report MWM performance in different groups of animals, at different ages. However, few studies report repeated-MWM performance testing in the same group of mice as they age. Nonetheless, a longitudinal-study design may be preferable to consider associative memory loss, common in neurodegenerative disorders such as Vascular Dementia and Alzheimer's disease.

A number of studies have shown that cognitive performance based on the latency-time to reach rescue platform during acquisition trialing of classic MWM, may be confounded because of age- or strain-associated differences in visual detection, acuity, or pattern recognition (Robinson et al., 2001; Võikar et al., 2001; Carman and Mactutus, 2002; Thifault et al., 2002; Brooks et al., 2005; Clapcote et al., 2005a,b; Brown and Wong, 2007). The classical MWM relies on processing of visual cues located external to the maze in order for the animal to orientate and locate a hidden platform. Whilst an intra-maze visible platform task may be used as a “control” for visual ability in classical MWM, the cued platform that signals location may not be an appropriate surrogate for extra-maze cues that are thought to guide classical MWM-behavior. Moreover, mice with compromised vision may move slower in spatial challenged settings, thereby confounding interpretation of cognitive function. To avoid misinterpretation of classic MWM performance because of differences in locomotor activity, Yanai and Endo (2016) recommended that for Senescent Accelerated Mouse Prone strains (SAMP) with an accelerated aging phenotype, classic MWM spatial performance be reported in the context of distance traveled, rather than latency time to reach rescue platform. However, of the many SAMP-focused studies, few report distance swum in MWM, or locomotor speed.

Olfactory sense is critical for a range of mouse behaviors, including navigation, foraging, escape, object recognition, and behavioral elements (Doty, 1986; Schellinck et al., 1993; Brennan and Keverne, 2004; Keverne, 2004; Restrepo et al., 2004; Kavaliers et al., 2005). Olfaction is a central element of mouse species behavior within their social domain and indeed, is the principal sensory component in this species for associative learning and memory. It is a reasonable proposition that olfactory cues may be preferable to visual cues in spatial awareness water-maze testing protocols such as MWM, particularly if there is uncertainty as to the visual acuity of the strain with increasing age. Studies by Wong and Brown (2006) reported that a discriminatory odor challenge developed by Schellinck et al. (2001, 2004) could be effective for exploring murine learning and behavior and considered strain differences in cognitive ability demonstrated by classical MWM vs. conditioned odor preference tasks.

Pivotal to understanding cognitive effects with aging are *in vivo* models that simulate what occurs in humans. An established and widely used line of mice that feature accelerated aging are the Senescence Accelerated Mouse Prone (SAMP) strains (Takeda, 1999). Three decades of research support the contention that of these, SAMP strain-8 (SAMP8) is an appropriate model for considering human-aging, because pathological traits are age-dependent and occur as a consequence of epigenetic changes with age and heightened glucocorticoid exposure that synergistically promote oxidative stress (Flood and Morley, 1998; Hosokawa, 2002; Chiba et al., 2009; Griñan-Ferré et al., 2016a; Grinan-Ferre et al., 2016b; Puigoriol-Illamola et al., 2018). SAMP8 mice show normal development, but thereafter, have early loss of reproductive function and tend to demonstrate early manifestation of neurodegenerative features including neuronal cell loss and a reduction in neurotransmitter release (Sureda et al., 2006; Pallàs, 2012; Bernstein et al., 2014).

SAMP8 mice have been widely assessed for behavioral disturbances and the literature was comprehensively reviewed (Yanai and Endo, 2016). The SAMP8 strain are suggested to have spatial learning impairments from 12 weeks of age and spatial memory deficits commencing from 16 weeks of age (Ikegami et al., 1992; Flood and Morley, 1998; Cheng et al., 2008). Passive and active avoidance disturbances are reported as early as 12 weeks (Flood and Morley, 1993; Miyamoto, 1997) in SAMP8 mice and object recognition may be compromised in older age SAMP8 mice (36 weeks) (del Valle et al., 2012). Collectively, SAMP8 mice have been indicated as a relevant non-transgenic model for late-onset Alzheimer's disease (AD).

Several studies have considered olfactory sensitivity in SAMP8 mice. Ueno et al. (2016) reported that the barrier properties of the olfactory bulb deteriorate in pre-clinical rodent models of dementia including SAMP8 mice, findings consistent with clinical studies that suggest deterioration in olfaction as amongst the earliest indicators of cognitive decline (Attems et al., 2015; Devanand et al., 2015; Marin et al., 2018). In SAMP8 mice, Soriano-Cantón et al. (2015) reported that the B1-neural stem cells of the mouse sub-ependymal zone, which supports ongoing production of the olfactory bulb interneurons, are transiently expanded in young SAMP8 mice relative control senescent resistant SAMR1 mice. However, thereafter there is premature loss of the B1 stem cells in SAMP8 mice and by extension, possibly accelerated loss of olfactory associated cognitive function. Consistent with the latter, Ohta et al. (Ohta et al., 2002) utilized two-sets of non-spatial odor-odor pair learning protocols and concluded that SAMP8 mice experience age-dependent deficits in learning and memory of inferential tasks, compared to senescent resistant control mice (SAMR1), Spatially-cued challenges are considered a useful indicator of hippocampal-dependent memory, hence spatial-olfaction testing protocols may be a particularly helpful in the context of understanding cognitive function in animal models of dementia, that ordinarily have significant olfactory associative experiences.

The primary objective of this study was to determine if a modified MWM challenge protocol that utilized combinatory olfactory and visual cues would better discriminate cognitive strain performance differences between SAMP8 and SAMR1 control mice, compared to classic MWM testing protocols. The olfactory-visual water maze challenge described in this study included the provision of the olfactory cue positioned on the rescue platform above the water during the acquisition testing phase. Training for the olfactory-visual water maze challenge was provided once-only at a baseline age of 6 weeks and then repeatedly assessed in the same mice for a total of 4 times up to 40 weeks of age. The longitudinal design was to consider potential changes in associative memory with aging and we deliberately studied mice preceding substantial frank cognitive deficits that are suggested in SAMP8 mice to develop at greater than ~50 weeks of age The olfactory-visual cue challenge indicated, was compared to the commonly adopted testing protocol for classic MWM protocols, which was to train and test once-only, but in mice of different ages, that being at either 6, 20, 30, or 40 weeks of age.

## Materials and methods

### Animals

Male Senescence Accelerated Mouse Prone-Strain 8 (SAMP8) and their age-matched controls, Senescence Accelerated Mouse Resistant-Strain 1 (SAMR1) mice were obtained from the Animal Resources Centre (Murdoch, Western Australia). Mice were assigned to either the classical MWM testing procedure, with single training/acquisition testing at either 6, 20, 30, or 40 weeks of age (*n* = 10–15 per age group, per strain). Alternatively, mice were assigned to the modified combined olfactory-visual cued water maze protocol, where the same mouse was trained and assessed at 6, followed by repeated acquisition trialing thereafter at 20, 30, and 40 weeks of age (*n* = 19 per strain). All procedures were conducted as per National Health & Medical Research Council approved methods (AEC_2014/27).

### Maze apparatus and training regime

#### Apparatus

The water maze apparatus was constructed as described previously (Mamo et al., 2017). The water maze apparatus consisted of a white circular pool (120 cm width x 60 cm height), a transparent rescue platform (10 cm in diameter × 30 cm in height) submerged 1 cm below the water surface for classical water maze trialing. Pool temperature was maintained at 26 ± 1°C. The latency, swim path and locomotor ability of each mouse during each trial was tracked and recorded (HVS Image Software; Buckingham, U. K).

#### Classical morris water maze

Prior to assessment, all mice were subjected to 2 days of training with a submerged rescue platform, marked with an intra-maze visual cue, a black flag centrally placed on the transparent platform extending 5 cm above the water surface. Each mouse was lowered into the water, facing the pool wall and allowed 90 s to navigate around the pool and find the platform. Once on the rescue platform, each mouse had to stay on the platform for 30 s. Each training day consisted of 10 trials starting semi-randomly chosen compass points (N, S, E, W), with ~10 min between each inter-trial interval (Vorhees and Williams, 2006).

**Figure 1:**
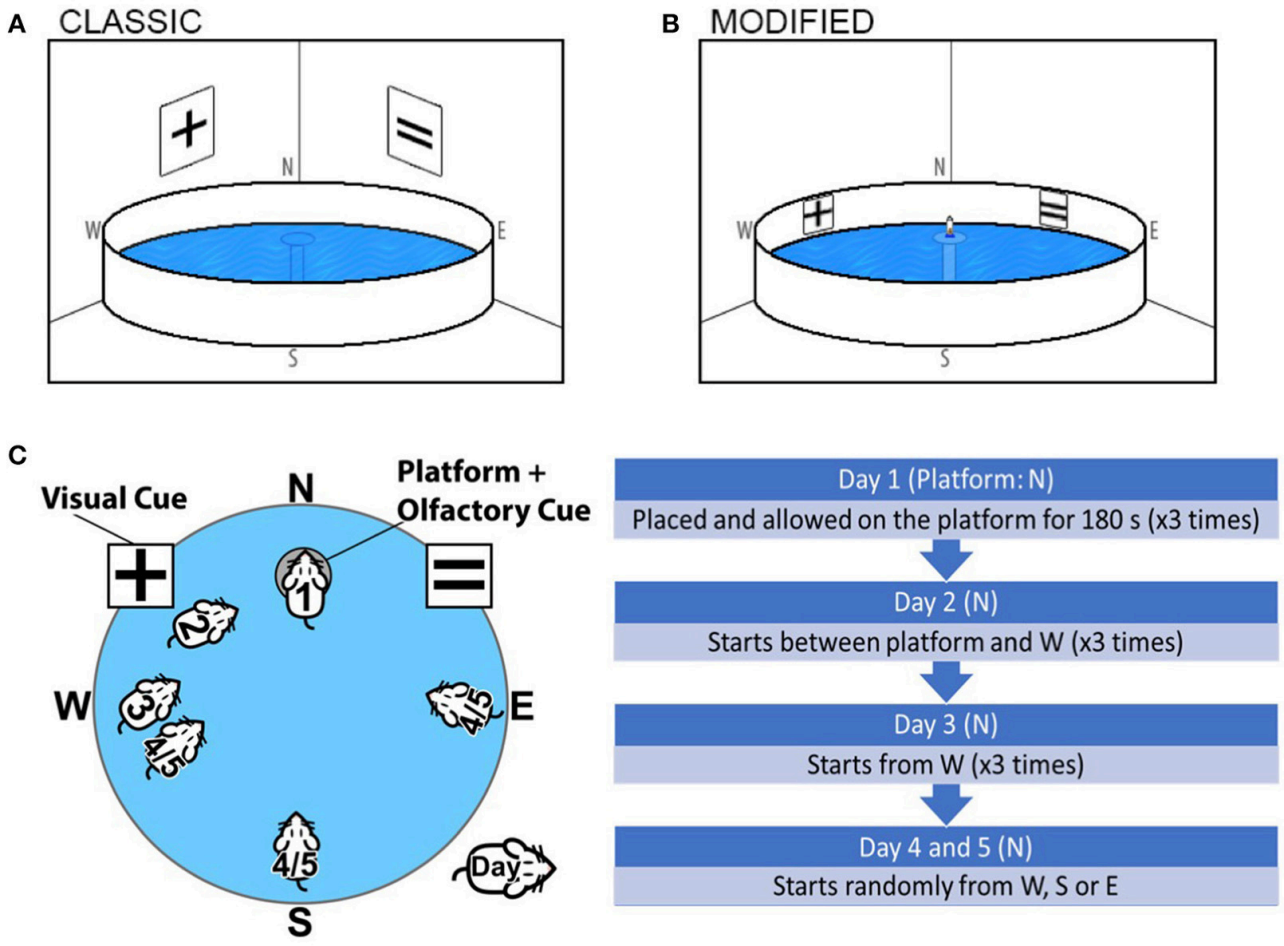
Graphical representation and comparison of the **(A)** Classical Morris Water Maze and **(B)** The Olfactory-Visual Water Maze. The Olfactory-Visual Water Maze is characterized by a combination of intra-maze cues (X and = symbols positioned on the X and Y position of the inner walls of the water tank) and a rescue platform positioned at N, 5 mm above the water surface fitted with a ventilated 50 mL Falcon tube containing freshly crushed chocolate and almond essence. **(C)** The chronology of the modified Visual-Olfactory Water maze regimen is indicated where all mice are subjected to a 5-day training/test regimen; 2 days of training (Day 1–2) followed by acquisition trials (Day 3–5). **(D)** Chronology of modified Visual-Olfactory Water maze.

Following training, the black flag on the platform was removed and each mouse went through a 5-day assessment with the hidden platform submerged 1 cm below the water surface. Large posters with distinct black shapes were also placed on the walls outside the pool as an extra-maze visual cue (Figure 1A). Identical to the training days, maximum swim time was set to 90 s and mice remained on the platform for 30 s. The platform remained in the same position, however, mice were placed in the pool from N, S, E, W positions, facing away from the platform. Each day consisted of 4 trials per mouse.

#### Olfactory-visual water maze

The protocol for the combined olfactory-visual cued water maze adopted a 5-day training/test regimen. In order to minimize the potential confounder of altered visual acuity with aging, alternate symbols of X and = were positioned on X and Y position of the inner wall of the water tank (Figure 1B). The rescue platform remained positioned above the water line (5 mm) throughout training and acquisition, fitted with a ventilated 50 mL Falcon tube (11 cm high, 3 cm in diameter) containing 3 g freshly crushed chocolate and 2 mL almond essence on gauze refreshed on each day of training. Throughout the training and acquisition trials, the platform was fixed in the N quadrant, and position of all cues remained at the same position.

#### Olfactory-visual water maze training

Prior to assessment, all mice were handled and trained for 2 days (Day 1–2). On Day 1, each mouse was placed on the olfactory-visual cued circular rescue platform located in the N quadrant of the pool, hosting the chocolate/almond charged ventilated Falcon tube. Mice were allowed to remain on the rescue platform for 180 s to familiarize and to orientate themselves with the maze and cues and showed significant interest in the Falcon tube. Of the few mice who left the platform during training (< 5%), these were gently guided back to the platform. On the second day of training (Day 2), each mouse was afforded three training trials. For trials 1, 2, and 3, mice were gently placed in the pool ~30, 60, and 90 cm away from the platform, respectively (in the W quadrant), always facing the rescue platform hosting the charged Falcon tube (Figure 1C). Mice had a maximum of 20, 25, and 30 s for the progressive training trials, respectively, to reach the platform and thereafter, allowed to sit on the platform for 180 s. Mice who failed to reach the platform within the allocated time were guided by the operator to the platform. Three familiarization (day 1) and three training trials (day 2) were separated by 2 h per trial.

#### Olfactory-visual water maze acquisition trials

Acquisition trials (Days 3–5) commenced 1 day following day 2 of training. The olfactory-visual cued platform was fixed in the same position (N quadrant). On Day 3, each mouse was released into the water facing ~60 cm distance (mid-W quadrant) facing the rescue platform hosting the charged Falcon tube (Figure 1C). During each trial, a maximum swim time was set to 60 s to reach the platform, and in failure to do so, the experimenter guided the mouse to the cued platform where the mouse was allowed to stay on the platform for 90 s. Three repeat trials were completed. The test conditions remained constant on Day 4 and 5, however, the starting point was varied between each trial (W, S, and E) (Figure 1C).

#### Longitudinal assessment of performance over the lifespan of SAM mice

Following training at 6 weeks of age, each animal was subjected to the exact 3-day acquisition protocol, as described above, upon reaching 6, 20, 30, and 40 weeks of age.

### Statistical analysis

For the cued learning and spatial acquisition trials for both the classic MWM and new olfactory-visual water maze protocols, the mean latency to reach the platform was calculated for each test day. For classical MWM, 10–12 male SAMP8 mice were studied at 6, 20, 30, and 40 weeks of age. For SAMR1 mice, 10 and up to 15 mice were studied. For the longitudinal olfactory-visual testing protocol, data was collected for *n* = 19 mice per strain (SAMP8 and SAMR1) to 40 weeks of age. Normalisation of variance was by way of the the Kolmogorov-Smirnov analysis and significance level is indicated on the figures.

## Results

**Figure 2:**
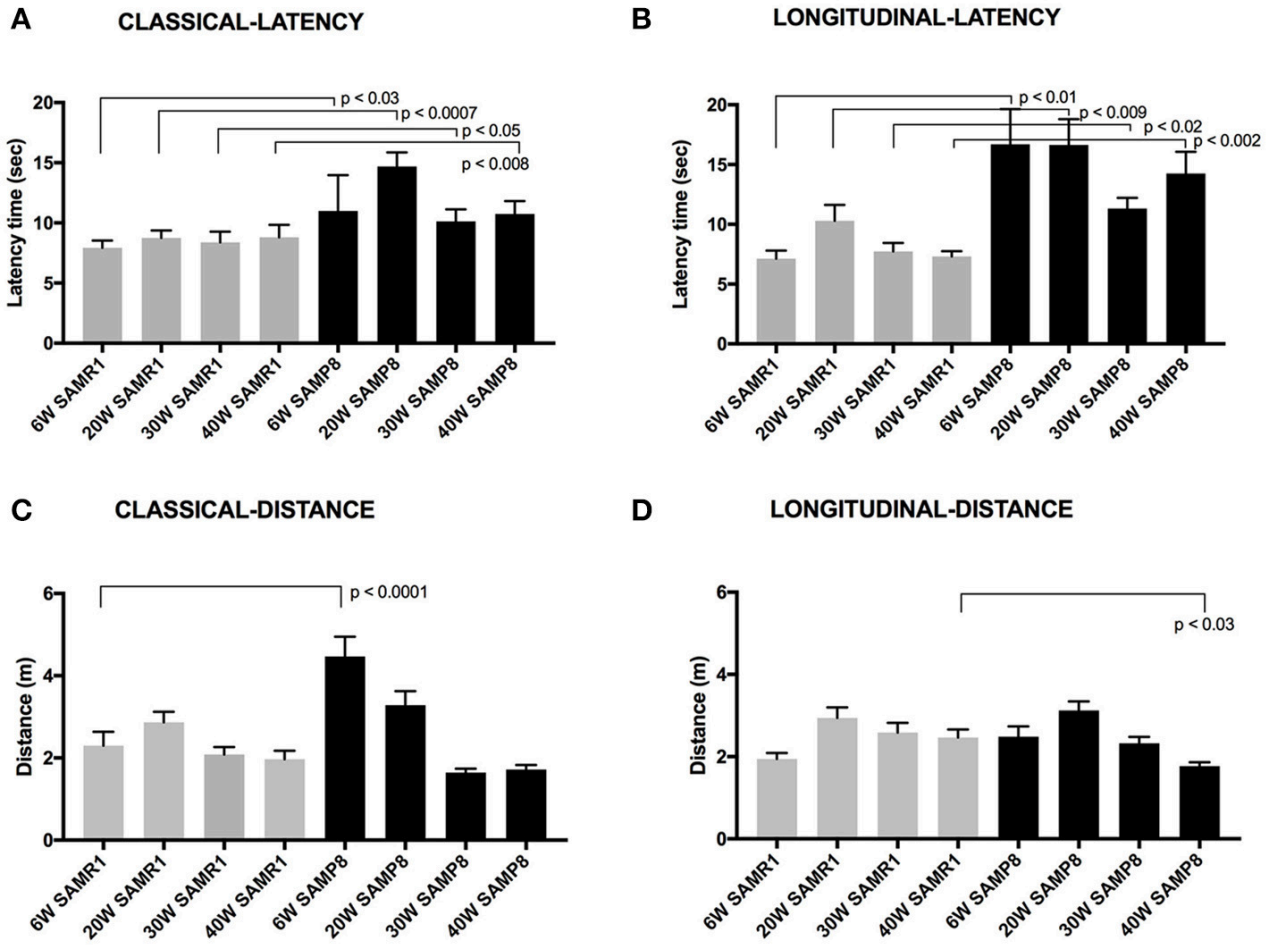
Maze latency times are presented (mean latency ± SEM for each test day) **(A)** The latency time for male SAMP8 and SAMR1 mice to reach a hidden rescue platform in classic MWM at 6, 20, 30, or 40 weeks of age. **(B)** Latency time to reach rescue platform in the olfactory-visual water maze in the same group of mice tested at the baseline age of 6-weeks and retested thereafter at 20, 30, and 40 weeks of age. The olfactory maze included a combination of intra-maze olfactory and visual cues positioned within the confines of the tank. **(C)** The distance swum for male SAMP8 and SAMR1 mice to reach a hidden rescue platform in classic MWM at 6, 20, 30, or 40 weeks of age. **(D)** Distance swum time to reach rescue platform in the olfactory-visual water maze in the same group of mice tested at the baseline age of 6-weeks and retested thereafter at 20, 30, and 40 weeks of age. Normalization of variance was by way of the Kolmogorov–Smirnov analysis and significance level is indicated.

In this study, cognitive performance of SAMP8 and SAMR1 was assessed using the classical extra-maze spatial-awareness MWM protocol determined in groups of mice trained and tested at either 6, 20, 30, or 40 weeks of age. Performance in classic MWM was compared to a combination olfactory-visual maze protocol assessed in a longitudinal lifespan context, which included training and testing at 6 weeks of age, followed by repeat acquisition testing of the same mice again at 20, 30, and 40 weeks of age. Figure 2 frame A, depicts time to the hidden rescue platform for the classic MWM in male mice trained and tested at one age, either 6, 20, 30, or 40 weeks. Performance in the classic MWM showed little variability within each age/strain, indicative of a carefully administered classic MWM protocol. In this study, the classic MWM demonstrates for the first time at 6 weeks of age, significant strain difference in latency-time to reach the hidden platform. The strain difference shown by the classical MWM in male mice at 6 weeks was also present at 20, 30, and 40 weeks of age, consistent with previous reports. However, in addition, we now show that when performance in classic MWM is assessed once but at different ages, there is no evidence of an age-associated divergence between SAMP8 and SAMR1 control mice up to 40 weeks of age.

To consider potential locomotor effects for classic MWM time-to-reach-platform measures indicated in Figure 2 frame A, the distance traveled to reach the hidden platform is also reported (Figure 2, frame C). The SAMP8 mice assessed at the earliest age of 6 weeks were found to have traveled ~2-fold more than age-matched control SAMR1 mice. Thereafter, there was progressive attenuation of the distance traveled in SAMP8 mice when trained and assessed at 20 weeks of age and with a further decline in distance traveled if assessed at 30 weeks of age. Indeed, the total distance traveled in classic MWM by SAMP8 mice at 30 and 40 weeks of age was comparable to SAMR1 controls of same age (Figure 2, frame C).

An olfactory-visual water maze protocol was developed to potentially capitalize on the dominant olfactory spatial-cue driver in rodent species. Moreover, the spatial olfactory-visual water maze was administered in a longitudinal context, because repeat measures in the same animals would notionally be more indicative of associative memory experience changes that are relevant to cognitive decline in human dementia disorders. Male SAMP8 and SAMR1 mice were trained and assessed at 6 weeks of age and thereafter, the same mice underwent repeated acquisition trialing at 20, 30, and 40 weeks of age. Figure 2, Frame B, depicts longitudinal time-to-reach rescue platform performance for each strain. At 6 weeks of age, SAMP8 mice took almost three times longer to reach the olfactory-visual rescue platform compared to age-matched SAMR1 mice. The findings are qualitatively consistent with that indicated for classic MWM at same age (Figure 2, frame A), however the strain differences were substantially more separated for the olfactory-spatial water maze challenge protocol. Interpretation of SAMP8 performance in the olfactory-visual water maze challenge without consideration of locomotor activity immediately suggest significant functional impairment in the SAMP8 mice as early as 6 seeks of age, however consideration of the distance traveled (Figure 2, frame D) shows that total actual distance swum by SAMP8 mice was similar to SAMR1 mice at 6 weeks of age. The slower time to reach the olfactory-visual rescue platform was therefore a consequence of either significantly slower processing of the olfactory-visual cue, or poorer locomotor functioning. The comparable distances swum by SAMP8 and SAMR1 mice in the olfactory-spatial water maze challenge at 6 weeks of age, completely contrasts with that indicated for classic MWM at 6 weeks, where SAMP8 mice had substantially greater swimming distance recorded than SAMR1 mice. Consistent strain differences in the latency time to reach the rescue platform in the olfactory-visual water maze challenge (Figure 2, frame B), but similar strain performances for the actual total distance swum (Figure 2, frame D) were realized in SAMP8 and SAMR1 mice when repeat tested at 20, 30, or 40 weeks of age. The longitudinal measures for either latency-time, or distance swum in the olfactory-visual water maze challenge, did not demonstrate and associative memory loss in SAMP8 mice to 40 weeks of age.

## Discussion

In rodents, odor processing involves processing within the sensory neuron to the olfactory bulb, decoding in the piriform cortex and thereafter, in downstream neurons in the hippocampus (Wilson and Stevenson, 2006). Some studies suggest olfactory challenges can be reliably utilized to assess long-term associative memory in mice (Schellinck et al., 2001) but this has never been explored directly. In this study, we compared from a young age of 6 weeks cognitive and motor performance of SAMP8 in an olfactory-visual spatial water maze task administered longitudinally. The spatial olfactory-visual water maze challenge affirms findings in classic MWM that time to reach a rescue a platform is delayed in SAMP8 mice compared to SAMR1 mice and that this strain difference is apparent from as young as 6 weeks of age. The markedly greater strain differential in latency time to reach the rescue platform in the olfactory-visual water maze challenge compared to the classic MWM, might be indicative of significant olfactory processing deficits in the SAMP8 strain. Consistent with the latter, Soriano-Cantón et al. (2015) reported a substantially reduced density of the mouse sub-ependymal zone in SAMP8 mice compared to SAMR1 at 2 months of age. In other studies, Ohta et al. (2002) reported age-associated learning and memory deficits in SAMP8 mice challenged with non-spatial odor-odor trialing. However, in Ohta's report, SAMP8 performance differences relative to SAMR1 mice were not realized until ~4 months of age. The olfactory-visual water maze challenge protocol, may discriminate olfactory deficits in SAMP8 mice as young as 6 weeks of age.

An alternative explanation for the marked strain differences in time-to-reach rescue platform indicated by the olfactory-visual water maze challenge is that the SAMP8 mice have locomotor deficits, taking longer to reach the rescue platform. However, performance of SAMP8 and control SAMR1 mice was also determined using a classical MWM protocol, that requires the rodent to find a hidden platform based on spatial cues alone, that are external to the maze tank. The classic MWM confirmed strain differences in time to reach rescue platform, however at 6 weeks of age, the total distance swum was twice that of SAMP8 mice tested for the first time at 6 weeks of age in the olfactory-visual water maze challenge (Figure 2, frame C vs. frame D). The paradoxical findings of distance swum for the olfactory-visual water maze challenge vs. the classic MWM in SAMP8 mice at 6 weeks of age, suggests that a locomotor defect was not responsible for the slower time-to-reach platform performance in the olfactory-visual water maze challenge protocol. Rather, strain differences in olfactory cue processing is more likely to explain the latency-time performance measures. The repeat test measures of latency time to reach rescue platform and total distance traveled in the olfactory-visual water maze challenge were consistent in SAMP8 mice when at 20, 30, or 40 weeks of age, suggesting no further age-associated decline in performance to 40 weeks of age (Figure 2, frames C and D).

A number of age-related changes in SAMP8 mice suggest them to be a good model for late-onset AD and indeed, reported to serve as a useful model for understanding basic cellular processes modified with aging that are likely to impact upon cognitive function. Odor deficits have been demonstrated early in AD patients (Albers et al., 2018) and are also present in genetic models of AD (Van Dijck et al., 2008; Young et al., 2009; Montgomery et al., 2011). This study adopted for the first time an integrated olfactory and visual cue protocol that is easily administered, shows limited variability within treatment, and enables longitudinal assessment with consideration of compensatory changes in visual and olfactory cue processing. The protocol is physiologically relevant for considering potential confounding factors often present in the classic MWM. To 40 weeks of age, utilizing intra-maze olfactory-visual cues during a water maze challenge, the present study suggests that strain differences in MWM performance may be confounded by olfactory processing deficits. The findings complement a vast body of literature in SAMP8 mice that demonstrate central nervous system physiological aberrations preceding loss of spatial and associative memory. Extension of the olfactory-visual maze testing protocol at older age and its relevance to physiological and pathological aberrations would be informative.

## Ethics statement

The Australian National Health and Medical Research Council accredited Curtin University Animal Ethic Committee approved the study design and protocols detailed in this manuscript (approval number AEC_2014/27). The process requires substantial information regarding the hypothesis, justification for the use of animals and number indicated, rationalization for experimental design, comprehensive details of experimental protocols and operating procedures, evidence of competency, and accredited animal handling techniques. Credibility of research team is also considered.

## Author contributions

VL contributed to design of study, collection of data, interpretation, and writing. RT, MA, ZD, LG, MH, NF, and MN contributed to design of study, interpretation, and writing. SC contributed to data collection and analysis. JM conceived the perspective, designed experiment, led interpretation of data, and writing of manuscript.

### Conflict of interest statement

The authors declare that the research was conducted in the absence of any commercial or financial relationships that could be construed as a potential conflict of interest.

## References

[B1] Albers M. W. Tabert M. H. Devanand D. P. (2018). Olfactory dysfunction as a predictor of neurodegenerative disease. Curr. Neurol. Neurosci. Rep. 6, 379–386. 16928347 10.1007/s11910-996-0018-7

[B2] Attems J. Walker L. Jellinger K. A. (2015). Olfaction and aging: a mini-review. Gerontology 61, 485–490. 10.1159/000381619 25968962

[B3] Bernstein L. R. Mackenzie A. C. Kraemer D. C. Morley J. E. Farr S. Chaffin C. L.. (2014). Shortened estrous cycle length, increased FSH levels, FSH variance, oocyte spindle aberrations, and early declining fertility in aging senescence-accelerated mouse prone-8 (SAMP8) mice: concomitant characteristics of human midlife female reproductive aging. Endocrinology. 155, 2287–2300. 10.1210/en.2013-2153 24654787

[B4] Brennan P. A. Keverne E. B. (2004). Something in the air? New insights into mammalian pheromones. Curr. Biol. 14, R81–R89. 10.1016/j.cub.2003.12.052 14738757

[B5] Brooks S. P. Pask T. Jones L. Dunnett S. B. (2005). Behavioural profiles of inbred mouse strains used as transgenic backgrounds. II: cognitive tests. Genes. Brain Behav. 4, 307–17. 10.1111/j.1601-183X.2004.00109.x 16011577

[B6] Brown R. E. Wong A. A. (2007). The influence of visual ability on learning and memory performance in 13 strains of mice. Learn Mem. 14, 134–144. 10.1101/lm.473907 17351136 PMC1838554

[B7] Carman H. M. Mactutus C. F. (2002). Proximal versus distal cue utilization in spatial navigation: the role of visual acuity? Neurobiol. Learn Mem. 78, 332–46. 12431421 10.1006/nlme.2002.4062

[B8] Cheng H. Yu J. Jiang Z. Zhang X. Liu C. Peng Y.. (2008). Acupuncture improves cognitive deficits and regulates the brain cell proliferation of SAMP8 mice. Neurosci. Lett. 432, 111–116. 10.1016/j.neulet.2007.12.009 18215464

[B9] Chiba Y. Shimada A. Kumagai N. Yoshikawa K. Ishii S. Furukawa A.. (2009). The senescence-accelerated mouse (SAM): a higher oxidative stress and age-dependent degenerative diseases model. Neurochem. Res. 34, 679–687. 10.1007/s11064-008-9812-8 18688709

[B10] Clapcote S. J. Lazar N. L. Bechard A. R. Roder J. C. (2005a). Effects of the *rd1* mutation and host strain on hippocampal learning in mice. Behav. Genet. 35, 591–601. 10.1007/s10519-005-5634-5 16184487

[B11] Clapcote S. J. Lazar N. L. Bechard A. R. Wood G. A. Roder J. C. (2005b). NIH Swiss and Black Swiss mice have retinal degeneration and performance deficits in cognitive tests. Comp. Med. 55, 310–316. 16158906

[B12] del Valle J. Bayod S. Camins A. Beas-Zárate C. Velázquez-Zamora D. A. González-Burgos I.. (2012). Dendritic spine abnormalities in hippocampal CA1 pyramidal neurons underlying memory deficits in the SAMP8 mouse model of Alzheimer's disease. J. Alzheimers Dis. 32, 233–240. 10.3233/JAD-2012-120718 22776969

[B13] Devanand D. P. Lee S. Manly J. Andrews H. Schupf N. Doty R. L.. (2015). Olfactory deficits predict cognitive decline and Alzheimer dementia in an urban community. Neurology. 84, 182–189. 10.1212/WNL.0000000000001132 25471394 PMC4336090

[B14] Doty R. L. (1986). Odor-guided behavior in mammals. Experientia 42, 257–271. 10.1007/BF01942506 3514263

[B15] Flood J. F. Morley J. E. (1993). Age-related changes in footshock avoidance acquisition and retention in senescence accelerated mouse (SAM). Neurobiol. Aging 14, 153–157. 10.1016/0197-4580(93)90091-O 8487918

[B16] Flood J. F. Morley J. E. (1998). Learning and memory in the SAMP8 mouse. Neurosci. Biobehav. Rev. 22, 1–20. 10.1016/S0149-7634(96)00063-2 9491937

[B17] Griñan-Ferré C. Palomera-Ávalos V. Puigoriol-Illamola D. Camins A. Porquet D. Plá V.. (2016a). Behaviour and cognitive changes correlated with hippocampal neuroinflammaging and neuronal markers in female SAMP8, a model of accelerated senescence. Exp. Gerontol. 80, 57–69. 10.1016/j.exger.2016.03.014 27094468

[B18] Grinan-Ferre C. Perez-Caceres D. Gutierrez-Zetina S. M. Camins A. Palomera-Avalos V. Ortuno-Sahagun D.. (2016b). Environmental enrichment improves behavior, cognition, and brain functional markers in young senescence-accelerated prone mice (SAMP8). 53, 2435–2450. 10.1007/s12035-015-9210-6 26014386

[B19] Hosokawa M. (2002). A higher oxidative status accelerates senescence and aggravates age-dependent disorders in SAMP strains of mice. Mech. Ageing Dev. 123, 1553–1561. 10.1016/S0047-6374(02)00091-X 12470893

[B20] Ikegami S. Shumiya S. Kawamura H. (1992). Age-related changes in radial-arm maze learning and basal forebrain cholinergic systems in senescence accelerated mice (SAM). Behav. Brain Res. 51, 15–22. 10.1016/S0166-4328(05)80307-9 1482543

[B21] Kavaliers M. Choleris E. Pfaff D. W. (2005). Recognition and avoidance of the odors of parasitized conspecifics and predators: differential genomic correlates. Neurosci. Biobehav. Rev. 29, 1347–1359. 10.1016/j.neubiorev.2005.04.011 16055189

[B22] Keverne E. B. (2004). Importance of olfactory and vomeronasal systems for male sexual function. Physiol. Behav. 83, 177–187. 10.1016/j.physbeh.2004.08.013 15488538

[B23] Mamo J. C. L. Lam V. Giles C. Coulson S. H. Fimognari N. Mooranian A. (2017). Antihypertensive agents do not prevent blood-brain barrier dysfunction and cognitive deficits in dietary-induced obese mice. Int. J. Obes. 41, 926–934. 10.1038/ijo.2017.57 28239165

[B24] Marin C. Vilas D. Langdon C. Alobid I. López-Chacón M. Haehner A.. (2018). Olfactory dysfunction in Neurodegenerative diseases. Curr. Allergy Asthma Rep. 18:42. 10.1007/s11882-018-0796-4 29904888

[B25] Miyamoto M. (1997). Characteristics of age-related behavioral changes in senescence-accelerated mouse SAMP8 and SAMP10. Exp. Gerontol. 32, 139–148. 10.1016/S0531-5565(96)00061-7 9088911

[B26] Montgomery K. S. Simmons R. K. Edwards G. III. Nicolle M. M. Gluck M. A. Myers C. E.. (2011). Novel age-dependent learning deficits in a mouse model of Alzheimer's disease: implications for translational research. Neurobiol. Aging 32, 1273–1285. 10.1016/j.neurobiolaging.2009.08.003 19720431 PMC4334376

[B27] Ohta A. Akiguchi I. Seriu N. Ohnishi K. Yagi H. Higuchi K.. (2002). Deterioration in learning and memory of inferential tasks for evaluation of transitivity and symmetry in aged SAMP8 mice. Hippocampus 12, 803–810. 10.1002/hipo.10046 12542231

[B28] Pallàs M. (2012). Senescence-accelerated mice P8: a tool to study brain aging and alzheimer's disease in a mouse model. ISRN Cell Biol. 2012, 1–12. 10.5402/2012/917167

[B29] Puigoriol-Illamola D. Griñán-Ferré C. Vasilopoulou F. Leiva R. Vázquez S. Pallàs M. (2018). 11β-HSD1 inhibition by RL-118 promotes autophagy and correlates with reduced oxidative stress and inflammation, enhancing cognitive performance in SAMP8 mouse model. Mol. Neurobiol. 10.1007/s12035-018-1026-8. [Epub ahead of print]. 29611102

[B30] Restrepo D. Arellano J. Oliva A. M. Schaefer M. L. Lin W. (2004). Emerging views on the distinct but related roles of the main and accessory olfactory systems in responsiveness to chemosensory signals in mice. Horm Behav. 46, 247–256. 10.1016/j.yhbeh.2004.02.009 15325226

[B31] Robinson L. Bridge H. Riedel G. (2001). Visual discrimination learning in the water maze: a novel test for visual acuity. Behav. Brain Res. 119, 77–84. 10.1016/S0166-4328(00)00334-X 11164528

[B32] Schellinck H. M. Arnold A. Rafuse V. F. (2004). Neural cell adhesion molecule (NCAM) null mice do not show a deficit in odour discrimination learning. Behav. Brain Res. 152, 327–334. 10.1016/j.bbr.2003.10.011 15196800

[B33] Schellinck H. M. Forestell C. A. LoLordo V. M. (2001). A simple and reliable test of olfactory learning and memory in mice. Chem. Sens. 26, 663–672. 10.1093/chemse/26.6.663 11473932

[B34] Schellinck H. M. Smyth C. Brown R. Wilkinson M. (1993). Odor-induced sexual maturation and expression of c-fos in the olfactory system of juvenile female mice. Brain Res. Dev. Brain Res. 74, 138–141. 10.1016/0165-3806(93)90094-Q 8403368

[B35] Soriano-Cantón R. Perez-Villalba A. Morante-Redolat J. M. Marqués-Torrejón M.Á. Pallás M. Pérez-Sánchez F.. (2015). Regulation of the p19(Arf)/p53 pathway by histone acetylation underlies neural stem cell behavior in senescence-prone SAMP8 mice. Aging Cell. 14, 453–462. 10.1111/acel.12328 25728253 PMC4406674

[B36] Sureda F. X. Gutierrez-Cuesta J. Romeu M. Mulero M. Canudas A. M. Camins A.. (2006). Changes in oxidative stress parameters and neurodegeneration markers in the brain of the senescence-accelerated mice SAMP-8. Exp. Gerontol. 41, 360–367. 10.1016/j.exger.2006.01.015 16542809

[B37] Takeda T. (1999). Senescence-accelerated mouse (SAM): a biogerontological resource in aging research. Neurobiol. Aging. 20, 105–110. 10.1016/S0197-4580(99)00008-1 10537019

[B38] Thifault S. Lalonde R. Sanon N. Hamet P. (2002). Comparisons between C57BL/6J and A/J mice in motor activity and coordination, hole-poking, and spatial learning. Brain Res. Bull. 58, 213–218. 10.1016/S0361-9230(02)00782-7 12127020

[B39] Ueno M. Chiba Y. Matsumoto K. Murakami R. Fujihara R. Kawauchi M.. (2016). Blood-brain barrier damage in vascular dementia. Neuropathology 36, 115–124. 10.1111/neup.12262 26607405

[B40] Van Dijck A. Vloeberghs E. Van Dam D. Staufenbiel M. De Deyn P. P. (2008). Evaluation of the APP23-model for Alzheimer's disease in the odour paired-associate test for hippocampus-dependent memory. Behav. Brain Res. 190, 147–151. 10.1016/j.bbr.2008.02.014 18359101

[B41] Võikar V. Kõks S. Vasar E. Rauvala H. (2001). Strain and gender differences in the behavior of mouse lines commonly used in transgenic studies. Physiol. Behav. 72, 271–281. 10.1016/S0031-9384(00)00405-4 11240006

[B42] Vorhees C. V. Williams M. T. (2006). Morris water maze: procedures for assessing spatial and related forms of learning and memory. Nat. Protoc. 1, 848–858. 10.1038/nprot.2006.116 17406317 PMC2895266

[B43] Wilson D. A. Stevenson R. J. (2006). Learning to Smell: Olfactory Perception from Neurobiology to Behavior. Baltimore, MD: The Johns Hopkins University Press, 2006.

[B44] Wong A. A. Brown R. E. (2006). Visual detection, pattern discrimination and visual acuity in 14 strains of mice. Genes Brain Behav. 5, 389–403. 10.1111/j.1601-183X.2005.00173.x 16879633

[B45] Yanai S. Endo S. (2016). Early onset of behavioral alterations in senescence-accelerated mouse prone 8 (SAMP8). Behav. Brain Res. 308, 187–195. 10.1016/j.bbr.2016.04.026 27093926

[B46] Young J. W. Sharkey J. Finlayson K. (2009). Progressive impairment in olfactory working memory in a mouse model of mild cognitive impairment. Neurobiol. Aging 30, 1430–1443. 10.1016/j.neurobiolaging.2007.11.018 18242780

